# Interleukin-33-Dependent Accumulation of Regulatory T Cells Mediates Pulmonary Epithelial Regeneration During Acute Respiratory Distress Syndrome

**DOI:** 10.3389/fimmu.2021.653803

**Published:** 2021-04-15

**Authors:** Wen Tan, Bohan Zhang, Xinpei Liu, Chaoji Zhang, Jianzhou Liu, Qi Miao

**Affiliations:** ^1^Department of Cardiac Surgery, Peking Union Medical College Hospital, Chinese Academy of Medical Sciences and Peking Union Medical College, Beijing, China; ^2^Center for Cardiac Intensive Care, Beijing Anzhen Hospital, Capital Medical University, Beijing, China

**Keywords:** regulatory T cells, acute respiratory distress syndrome, IL-33:ST2 axis, pulmonary regeneration, lung repair

## Abstract

Acute respiratory distress syndrome (ARDS) triggered mostly by infection, is a syndrome that involves respiratory failure. ARDS induces strong local infiltration of regulatory T cells (Treg cells) in the lungs, and Treg cells were recently highlighted as being related to the repair of various tissue. However, at present, there is still a lack of adequate evidence showing the impact of Treg cells on pulmonary regeneration during ARDS. Here, we verified that Treg cells are strongly induced in ARDS mice and Treg depletion results in impaired lung repair. Moreover, Treg cells show high expression of ST2, a cellular receptor for the tissue alarmin IL-33, which is strongly upregulated in the lung during ARDS. In addition, we demonstrated that IL-33 signaling is crucial for Treg cell accumulation, and ST2-blocked mice show a decrease in the Treg cell population. Critically, transfer of exogenous IL-33 into Treg depleted mice restored Treg cells and facilitated lung regeneration by promoting alveolar type II cell (AEC2) recovery in ARDS, with elevated neutrophils infiltration and upregulated TGF-β1 release. These results emphasized the importance of IL-33 in accelerating the expansion of pulmonary Treg cells and promoting their activity to mediate pulmonary epithelial regeneration during ARDS in a TGF-β1-dependent manner.

## Introduction

Acute respiratory distress syndrome (ARDS), which is triggered mostly by infection, has hallmarks that include expiratory dyspnea and progressive hypoxemia, and the pivotal precipitating events are activation of the inflammatory micro-environment and diffuse alveolar damage (DAD). ARDS is associated with high mortality and a poor prognosis, an incidence of 78.9 per 100,000 persons in the US every year, approximately 156,000 people died of ARDS from 1999 to 2013 (1).

The cardinal pathophysiological features of ARDS are dysregulated inflammation and disruption of alveolar-capillary barrier. Upon damage, the lung immediately mounts a regenerative process orchestrated by intricate immune interaction. Extracellular matrix (ECM) remodeling, and epithelial-mesenchymal transition (EMT) (2, 3) was once determined to involve in pulmonary repair. In addition, epithelial repair, especially alveolar epithelial regeneration also acts a significant part. Alveolar epithelial regeneration, a process initiated by the proliferation and migration of endogenous progenitor alveolar type II cells (AEC2s), followed by their differentiation into alveolar type I cells (AEC1s) and restoration of epithelial barrier function, is still the focus of ongoing investigation. Previous study suggested a critical contribution of Treg cells to pulmonary repair *via* cross-talk between the adaptive and innate immune systems (4, 5). Moreover, the upregulation of Treg cells was not only correlated with the induction of immune tolerance (6), but also pivotal in restricting damage and coordinating the repair process.

Regulatory T cells (Treg cells), a unique population of lymphocytes, play an essential role in immune homeostasis. Classically, they are involved in various immune responses and suppress overwhelming immunity in several conditions, such as Th1-mediated colitis, sepsis, systemic lupus erythematosus (SLE), and transplant tolerance (7). Recent studies have verified that Treg cells are involved in non-immunological activity, for example, accelerating regeneration after muscle injury (8), enhancing healing after myocardial infarction, and improving skin barrier repair (9), by exerting direct/indirect effects on their progenitors or macrophage activity (10, 11). Certain local Treg cells, especially those in adipose tissue, muscle, or the intestines (8, 12, 13), express high amounts of the IL-33 receptor ST2 and require IL-33 for their regeneration, maintenance, or suppressive function.

Until now, the role of pulmonary Treg cells in the pathophysiology of ARDS has not been fully elucidated. Moreover, there has been limited evidence of the direct association between Treg cells and pulmonary epithelial regeneration in ARDS.

Here, we established a mouse model of LPS-induced ARDS to address whether population alteration or functional variation of Treg cells affected lung epithelial regeneration in mice. In addition, we explored endogenous cytokines that play critical roles in the process. We explicitly found dynamic Treg cell accumulation in inflamed lungs in LPS-induced ARDS, uncovered the significance of the interleukin (IL)-33:ST2 axis in expanding pulmonary Treg cell population. We exploited this axis to facilitate pulmonary repair in mice and revealed an association between IL-33-producing AEC2s, Treg cells and lung epithelial regeneration.

## Materials and Methods

### Animals

Adult male BALB/c mice aged 6–8 weeks were purchased from Beijing Vital River Laboratory Animal Technology Corporation and fed in the Animal Center of Peking Union Medical College Hospital (Beijing, China). All animals were kept in a specific-pathogen-free environment and maintained on standard mouse chow at an environmental temperature of 22–24°C with 12-h light and 12-h dark cycles.

Male mice were randomly allocated into several groups as follows: sham group, LPS-12-h group (L12h), LPS-1-day group (L1), LPS-2-day group (L2), LPS-4-day group (L4), LPS-7-day group (L7), LPS-9-day group (L9), LPS-11-day group (L11), and LPS-14-day group (L14). All mice were anesthetized with an intraperitoneal injection of 2% pentobarbital sodium (45 mg/kg body weight), and then, the mice received an intratracheal instillation of LPS (from *Escherichia coli* serotype O55:B5; Sigma-Aldrich Co, St. Louis, MO, USA) at a dose of 3 mg/kg. The sham group received only sterile saline (1.5 ml/kg).

This study was conducted in accordance with the National Institutes of Health Guide for the Care and Use of Laboratory Animals and the Animal Management Rules of the Chinese Ministry of Health. All experiments were approved by the Animal Care Committee of Peking Union Medical College.

### Treg Depletion

To deplete regulatory T cells, mice were intraperitoneally injected with 100 µg of anti-CD25 antibody (PC61; Biolegend, San Diego, CA, USA) 10 days before LPS exposure, and the treatment was repeated every 7 days for continuous Treg depletion. IgG was used as a control. Male mice were divided into four subgroups: Saline+IgG, Saline+anti-CD25, Lipopolysaccharide (LPS)+IgG, and LPS+anti-CD25. Then, the mice were sacrificed at 4 days.

### Assessment of Population Dynamics

To block lymphocyte migration from the peripheral lymphoid organs, the mice were treated with an S1P1 receptor agonist. FTY720 (25 mg/kg; Cayman Chemical, Ann Arbor, MI, USA) was i.p. injected prior to injury and daily thereafter. For quantification of T cell proliferation *in vivo*, 1 mg of EdU was i.p. injected, and 48 h later, Treg cells were processed for detection by the Click-iT plus EdU kit according to the manufacturer’s protocol (Molecular Probes, Waltham, MA, USA).

### ST2-Fc or IL-33 Treatment

Recombinant mouse ST2-Fc chimera protein (5 µg/mouse; R&D, Minneapolis, MN, USA) was administered i.p. 24 h before and after LPS challenge. The same amount of recombinant human IgG1 Fc (R&D, Minneapolis, MN, USA) was used as a control.

Recombinant mouse IL-33 (2 mg; Biolegend, San Diego, CA, USA) was administered *via* i.p. injection. IL-33 administration was performed the day prior to and the day after injury (8).

### Isolation of Lung and Spleen Cells

The right lung was removed, dissected into small sections, and incubated at 37°C in RPMI 1640 medium containing 1 mg/ml collagenase I and 50 µg/ml DNase (Invitrogen, San Diego, CA, USA) for 1 h. Then, the tissues were passed through a 70-µm nylon cell strainer (BD, Franklin Lakes, NJ, USA). The spleen was collected, ground, and mechanically dissociated in cold PBS. Samples were centrifuged at 300 g for 6 min at 4°C, washed, and resuspended in PBS after lysis of RBCs.

### Flow Cytometry

Lung and spleen cell staining was performed with CD16/CD32 Fc, CD4, CD25, CD31, CD326, CD45, F4/80 (Biolegend, San Diego, CA, USA), Ly6C, Ly6G, and CD11b (eBioscience, San Diego, CA, USA). Cells were fixed and permeabilized using a fixation/permeabilization kit (eBioscience, San Diego, CA, USA) according to the manufacturer’s instructions. Then, cells were stained for 30 min at 4°C with Foxp3 (Biolegend, San Diego, CA, USA) or Ki-67 (eBioscience, San Diego, CA, USA). The stained cells were washed twice and resuspended in 4% paraformaldehyde. Analysis of cell marker expression was performed using an Accuri C6 instrument (BD, Franklin Lakes, NJ, USA). Data were analyzed with FlowJo software.

### Transcript Analyses

For RNA-Seq analysis on the whole lung, the tissue was flash-frozen in liquid nitrogen and homogenized in TRIzol (Invitrogen, San Diego, CA, USA) before RNA extraction. Transcript analyses were performed on four samples. mRNA was purified from total RNA using poly-T oligo-attached magnetic beads. After fragmentation, reverse transcription and cluster generation, the library preparations were generated using NEBNext^®^ UltraTM RNA Library Prep Kit for Illumina^®^ (NEB, Ipswich, MA, USA) following manufacturer’s recommendations, and were sequenced on an illumine Hiseq Platform. Raw reads were collected and mapped to the reference genome, and then qualified by gene expression level. The Treg cell up/down signatures, AEC1 and AEC2 up/down signatures were developed and validated in a previous study (8, 14–16).

### Histopathological Analysis

The middle lobe of the right lung was fixed in 4% paraformaldehyde. The tissue was completely embedded in paraffin, cut into small sections, stained with hematoxylin and eosin, and measured by optical microscopy.

For immunofluorescence staining of mouse lungs, paraffin slides were dewaxed and dewatered, followed by permeabilization with 0.5% Triton X-100 for 10 min. Endogenous peroxidase blocking solution and goat serum were used to reduce the background. Then, the cells were stained with donkey polyclonal anti-human IL-33 (1:50, R&D, Minneapolis, MN, USA), rabbit polyclonal anti-mouse EpCAM (1:50, Abcam, Shanghai, China), anti-mouse SFTPC (1:50, Abcam, Shanghai, China), and anti-mouse CD31 (1:50, Abcam, Shanghai, China). Images were acquired with an Olympus Fluoview confocal microscope.

IL-33-positive nuclei were quantified automatically as the fraction of DAPI+ structures exhibiting IL-33 staining using ImageJ. The following settings were used: type-8 bit, invert, subtract background, threshold (80, 255), watershed, and analyze particles (size = 50, Infinity circularity = 0.1–1.00).

### RNA Extraction and Real-Time PCR

According to the manufacturer’s instructions, total RNA was collected from lung homogenates using the Eastep^®^ Super Total RNA Extraction Kit (Promega, Madison, WI, USA). Total RNA (1 μg) was reverse transcribed to cDNA using GoScript Reverse Transcriptase (Promega, Madison, WI, USA). Real-time quantitative PCR (qPCR) was performed using GoTaq qPCR mix (Promega, Madison, WI, USA) on the Applied Biosystems 7500 Fast system (Applied Biosystems, Waltham, MA, USA). The relative expression levels of target genes were quantified using the ΔΔCt method and normalized to the GAPDH gene, as described previously. The primer sequences are shown in **Table 1**.

**Table 1:** Primer sequences applied in real-time PCR.

Il33	Forward 5’-3’	CCTGCCTCCCTGAGTACATACA
	Reverse 5’-3’	CTTCTTCCCATCCACACCGT
GAPDH	Forward 5’-3’	AGGTCGGTGTGAACGGATTTG
	Reverse 5’-3’	GGGGTCGTTGATGGCAACA

### Western Blot Analysis

Lung tissues were collected and homogenized. Approximately 40 µg of protein was subjected to 10% sodium dodecyl sulfate polyacrylamide gel electrophoresis (SDS-PAGE). Then, the protein was transferred to 0.45 µM polyvinylidene fluoride (PVDF) membranes (Millipore, Schwalbach, Germany). The membranes were blocked with 5% non-fat powdered milk in Tris-buffered saline plus Tween 20 (TBST) and further incubated at 4°C overnight with primary antibodies against IL-33 (1:1,000, R&D, Minneapolis, MN, USA), ST2 (1:1,000, Thermo, Waltham, MA, USA), E-cadherin (1:1,000; Abcam, Shanghai, China), AQP5 (1:1,000; Abcam, Shanghai, China), EpCAM (1:1,000; Abcam, Shanghai, China), Sftpc (1:1,000, Abcam, Shanghai, China), and β-actin (1:1,000; Abcam, Shanghai, China) at room temperature. After washing, proteins were detected with ECL plus reagent (Millipore, Burlington, MA, USA). Western blots were analyzed using ImageJ software (National Institutes of Health, Bethesda, MD).

### Bronchoalveolar Lavage

The mice were sacrificed, and bronchoalveolar lavage fluid (BALF) was collected by lavage of the left lung. BALF was centrifuged for 10 min at 300 g. The supernatant was removed and stored at −80°C for further detection. Then, red blood cell lysis buffer was added to the pellet to remove the red blood cells. Total BALF cells were counted using a hematocytometer. The remaining BALF cells were stained with Wright-Giemsa staining. Differential leukocyte counts were measured and observed by optical microscopy. Neutrophil and monocyte-macrophages were detected in a population of 200 cells using standard morphological criteria.

### Pulmonary Edema

We assess the pulmonary edema using the protein in BALF and wet/dry weight ratio. The BALF protein concentration was determined by the BCA Protein Assay Kit (Thermo Fisher Scientific, San Diego, CA, USA). To evaluate wet/dry ratio, the upper lobes of right lung were harvested and weighed, then placed in an oven at a temperature of 60°C for 5 days until the weight of lung tissues no longer changed. The dry lungs were weighed and wet/dry ratio was calculated.

### Accession Numbers

RNAseq data are available from the National Center for Biotechnology Information under Sequence Read Archive (SRA) submission: SUB8338323 (https://submit.ncbi.nlm.nih.gov/subs/sra/SUB8338323/overview), SUB8333637 (https://submit.ncbi.nlm.nih.gov/subs/sra/SUB8333637/overview).

### ELISA Kit and Multiplex Immunoassays

Levels of interleukin (IL)-6, interleukin (IL)-8, and transforming growth factor (TGF)-β1 in BALF were determined using ELISA kit to assess the pulmonary inflammation during the process of resolution.

Secreted soluble protein in BALF was detected by ProcartaPlexTM multiplex immunoassays (Thermo Fisher Scientific, San Diego, CA, USA). In brief, multiple magnetic beads were mixed with 50 µl BALF supernatant and shaken on a plate shaker for 2 h at room temperature. Then, 25 µl of detection antibodies, 50 µl of SA-PE was successively added to the solution for 30 min. The beads were carefully washed and resuspended. Samples were collected by a LuminexTM instrument (Thermo Fisher Scientific, San Diego, CA, USA).

### Statistical Analysis

Data are presented as the mean ± S.D. All data were analyzed using one-way analysis of variance with the Bonferroni *post hoc* test for multiple t-tests. A value of p < 0.05 was considered statistically significant.

## Results

### Lung Injury Exhibited a Dynamic Variation Within 2 Weeks During LPS-Induced ARDS

**Figure 1:**
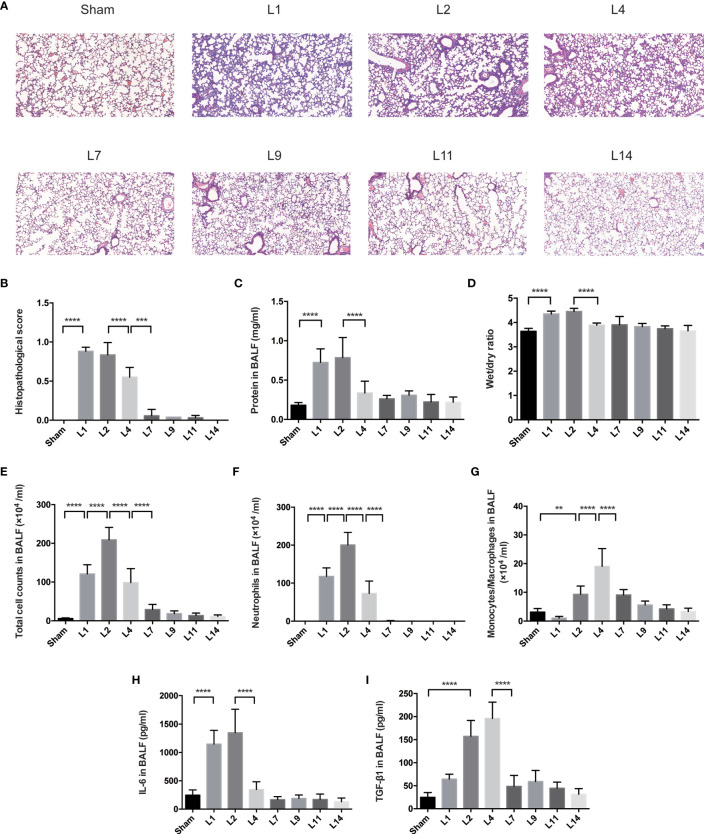
Lung injury exhibited a dynamic variation within 2 weeks during LPS-induced ARDS. **(A)** Histopathological staining. Representative HE staining of lung sections are shown 1, 2, 4, 7, 9, 11, 14 days after LPS-induced ARDS. Original magnification = 100×, scale bar represents 100 μm. **(B)** Histopathological scores of **(A)**. Lung injury was evaluated on a scale of 0–2 for each of the following criteria: i) neutrophils in the alveolar space; ii) neutrophils in the interstitial space; iii) hyaline membranes; iv) proteinaceous debris; v) alveolar septal thickening. The final histopathological scores was as follows: score = [20*(i)+14*(ii)+7*(iii)+7*(iv)+2*(v)]/(number of fields *100) **(C)** The protein in BALF. **(D)** The wet/dry ratio of lung tissue. **(E)** Total cell counts in BALF. **(F)** Neutrophils in BALF. **(G)** Monocytes/Macrophages in BALF. **(H)** Secretion of cytokine IL-6 in BALF. **(I)** Secretion of cytokine TGF-β1 in BALF. For all panels: mean ± S.D. **p ≤ 0.01; ***p ≤ 0.001; and ****p ≤ 0.0001 for one-way analysis of variance with the Bonferroni *post hoc* test for multiple t-tests. A value of p < 0.05 was considered statistically significant. n = 6–8 mice per group.

Pulmonary histopathology revealed apparent neutrophil infiltration in both alveolar and interstitial space on day 1–2 after LPS administration, with heightened pulmonary edema, increased alveolar septum, even interstitial thickening. By day 4–7, lung histopathology had returned to basically normal state (**Figures 1A, B**).

The protein in BALF and wet/dry ratio were combined to assess the pulmonary permeability. During LPS-induced ARDS, the protein in BALF and wet/dry ratio were remained high on day 1–2, declined on day 4, then returned to baseline (**Figures 1C, D**). The cells infiltration in the alveolar space were also used to predict the lung inflammation. After LPS administration, the total cell and neutrophil counts in BALF remarkably increased on day 1, peaked on day 2, followed by an obvious reduction on day 4, finally generally normalized after 7 days’ recovery (**Figures 1E, F**). However, the variation of monocytes/macrophages counts decreased on day 1, then increased until it peaked on day 4 (**Figure 1G**).

Various inflammatory-related cytokines were involved in ARDS. The release of IL-6 in BALF peaked at day 2, then decreased in the following days until it came back to baseline (**Figure 1H**). Reversely, the levels of TGF-β were up-regulated on day 2, maintain highest on day 4, followed by a significant attenuation on day 7 (**Figure 1I**).

Indications of LPS-induced lung injury, including pulmonary histopathology, the protein in BALF, wet/dry ratio, total cell counts and differential counts in BALF, levels of pro-inflammatory cytokines (IL-6), and anti-inflammatory (TGF-β1), manifested similar degrees of injury peaked on day 1–2, followed by almost complete recovery over 4–7 days. Therefore, it is demonstrated that our murine model of ARDS represents a powerful experimental tool to further investigate the resolving and reparative processes of LPS-induced ARDS.

### Treg Cells Accumulate in LPS-Induced ARDS

**Figure 2:**
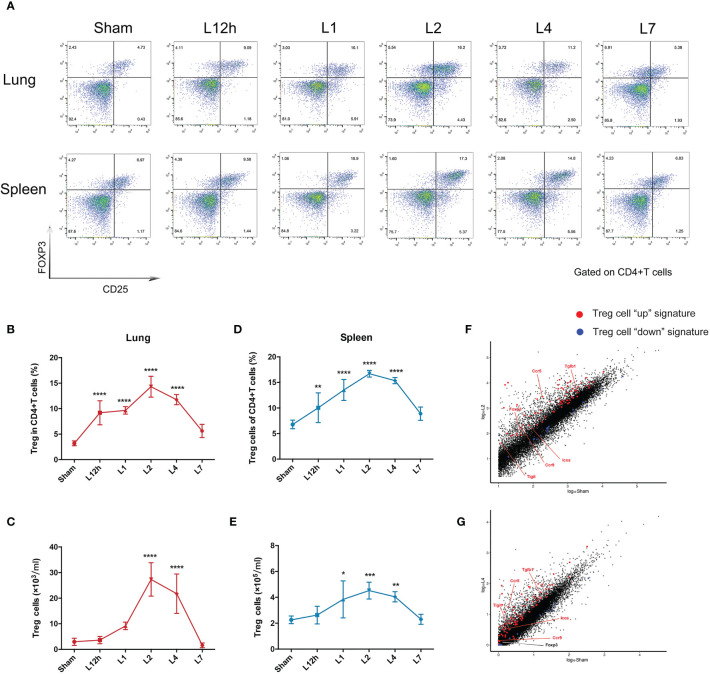
Treg cells accumulate in LPS-induced acute respiratory distress syndrome. BALB/c mice were intratracheally injected with LPS. The experiment was repeated twice. **(A)** Cytofluorometric dot plots of Tregs within days after injury. Numbers depict the fraction of CD4+ T cells within the designated gate. **(B)** Summary data for the fraction of Treg cells in the lungs depicted in **(A)**. **(C)** Summary data for the number of Treg cells in the lungs depicted in **(A)**. **(D)** Summary data for the fraction of Tregs in the spleen depicted in **(A)**. **(E)** Summary data for the number of Treg cells in the spleen depicted in **(A)**. **(F, G)** RNA-seq analysis. Normalized expression values for Treg cells **(F)** on day 2 and **(G)** on day 4 in the lung after LPS injury. Treg cell “up” (red) and “down” (blue) signature transcripts overlain, with some key Treg cell up genes highlighted. For all panels: mean ± S.D. *p ≤ 0.05; **p ≤ 0.01; ***p ≤ 0.001; and ****p ≤ 0.0001 for one-way analysis of variance with the Bonferroni post hoc test for multiple t-tests. A value of p < 0.05 was considered statistically significant. n = 6 mice per group.

Treg cells represent approximately 5% of the CD4+ T cell compartment in uninjured lungs of BALB/c mice. ARDS generated by intratracheal administration of LPS in mice leads to the dynamic accumulation of Treg cell populations within 1 week. The percentage of pulmonary Treg cells significantly increased 12 h after LPS administration, and the number of Treg cells remained highest on day 2, followed by a slight decrease on day 4. After 7 days of recovery, the number of pulmonary Treg cells was essentially normalized (**Figures 2A–C**). In addition, despite the pulmonary injury, the increase in splenic Treg cells showed similar dynamic variations (**Figures 2A, D, E**). Treg cells in the spleen and lung exhibited parallel changes, and the disturbance in Treg cells recovered within 1 week in the lungs and spleen. As day 2 and day 4 were two landmark timepoints, we chose them for following exploration.

The Treg cells in the inflamed lung could be verified by the fact that they displayed large numbers of typical diagnostic markers, for example, Foxp3. They also obviously expressed the characteristic Treg cell “up” signatures (8) (**Table S1**), according to RNA-seq analysis of the lung 2 days or 4 days after LPS administration when compared with the sham group. The fold changes of Treg cell “up” signatures observed after 2 days were more significant than those observed after 4 days **(****Figures 2F, G**).

### Treg Depletion Damages Pulmonary Epithelial Regeneration

Next, we sought to identify the role of Treg cells in LPS-induced ARDS. Proliferation of the alveolar epithelium or endothelium is an easily measurable event that leads to repair; it needs several hours to take place and at least approximately 2 days to be significant (17). In addition, it was reported that wound repair in the airways begins by 15–24 h and continues for days to weeks (18). Therefore, combined with our preliminary evidence, the process of pulmonary repair does not peak at an early stage after injury, and we chose the 4-day time point after LPS-induced lung injury for investigation.

**Figure 3:**
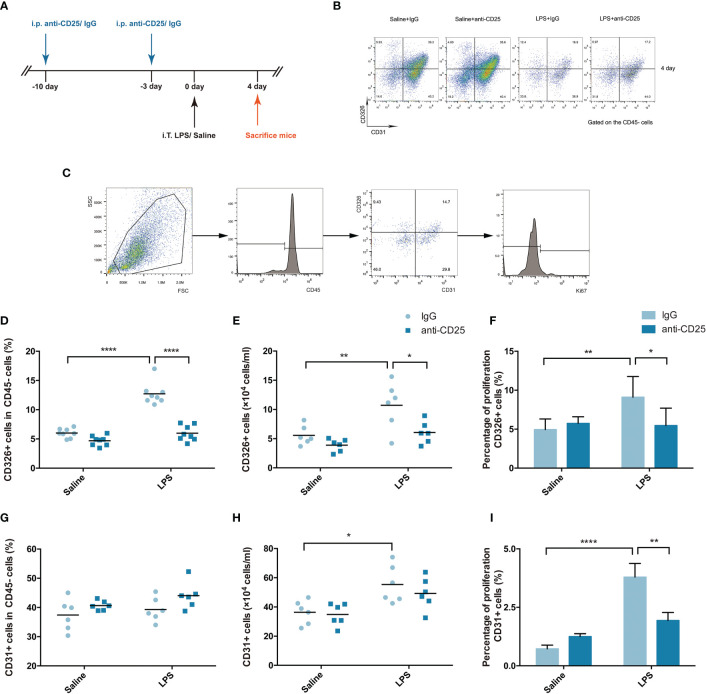
Treg depletion damages pulmonary epithelial regeneration. To deplete Treg cells, BALB/c mice were treated with an i.p. injection of anti-CD25 antibody on day 10 before LPS administration and 7 days thereafter. The mice were sacrificed 4 days after LPS exposure. **(A)** Schematic diagram of the experimental design. **(B)** Cytofluorometric dot plots of CD45−CD326+CD31− epithelial cells or CD45−CD326−CD31+ endothelial cells 4 days after injury. Numbers depict the fraction of CD45− cells within the designated gate. **(C)** Gating strategy to analyze the proliferation of epithelial cells/endothelial cells. Single-cell suspensions were prepared from injured or uninjured lungs, and Ki67+ epithelial cells were detected by flow cytometry. **(D)** Fraction of pulmonary epithelial cells (CD45−CD326+CD31−) in lung suspensions. **(E)** Number of pulmonary epithelial cells (CD45−CD326+CD31−) in lung suspensions. **(F)** Flow cytometric analysis of the proliferation of pulmonary epithelial cells. Proliferation was monitored by the nuclear marker Ki67. **(G)** Fraction of pulmonary endothelial cells (CD45−CD326−CD31+) in lung suspensions. **(H)** Number of pulmonary endothelial cells (CD45−CD326−CD31+) in lung suspensions. **(I)** Flow cytometric analysis of the proliferation of pulmonary endothelial cells. For all panels: mean ± S.D. *p ≤ 0.05; **p ≤ 0.01; and ****p ≤ 0.0001 for one-way analysis of variance with the Bonferroni post hoc test for multiple t-tests. A value of p < 0.05 was considered statistically significant. n = 6 mice per group.

A Treg-depleted mouse model was established by injection of anti-CD25 antibody every 7 days (**Figure 3A**), and the percentage of CD4+ effector T cells was not affected after Treg depletion in our previous study (19). Multicolor flow cytometry was used to specifically identify the pulmonary epithelium or endothelium in single-lung-cell suspensions, similar to previously reported methods (**Figures 3B, C**) (20). In our study, the fraction and number of epithelial cells were decreased in the Treg depleted mice after LPS (**Figures 3D, E**). Moreover, epithelial proliferation was also impaired when mice suffering from Treg depletion during ARDS (**Figure 3F**). However, the percentage or number of endothelial cells were not of significant difference (**Figures 3G, H**), albeit endothelial proliferation was damaged in Treg depleted mice after LPS administration (**Figure 3I**). Our data highly presented that Treg depletion devastated pulmonary regeneration by interfering with the epithelium.

### LPS-Induced ARDS Triggers the Release of IL-33 From the Inflamed Lung

To explore the central endogenous cytokines involved in LPS-induced ARDS, we performed mouse transcriptome sequencing (RNA-Seq analysis) to comprehensively understand the gene expression profile of inflamed lungs in LPS-induced ARDS. A total of 11,601 genes were identified to be significantly up- or downregulated (padj <0.05) in the inflamed lung in the L2 group compared with the sham group (**Table S2**). Among those differentially regulated genes, we focused on cytokines, especially inflammatory cytokines, that were remarkably upregulated. Considering the intrinsic basis of LPS-induced ARDS is respiratory infection followed by subsequent inflammation, we investigated KEGG enrichment of respiratory infection and cytokine-cytokine interactions for clue. Respiratory infection included 118 significant genes, and 124 significant genes were involved in cytokine-cytokine interactions. Searching for the overlap between these two enriched categories can provide us with available information about key cytokines.

**Figure 4:**
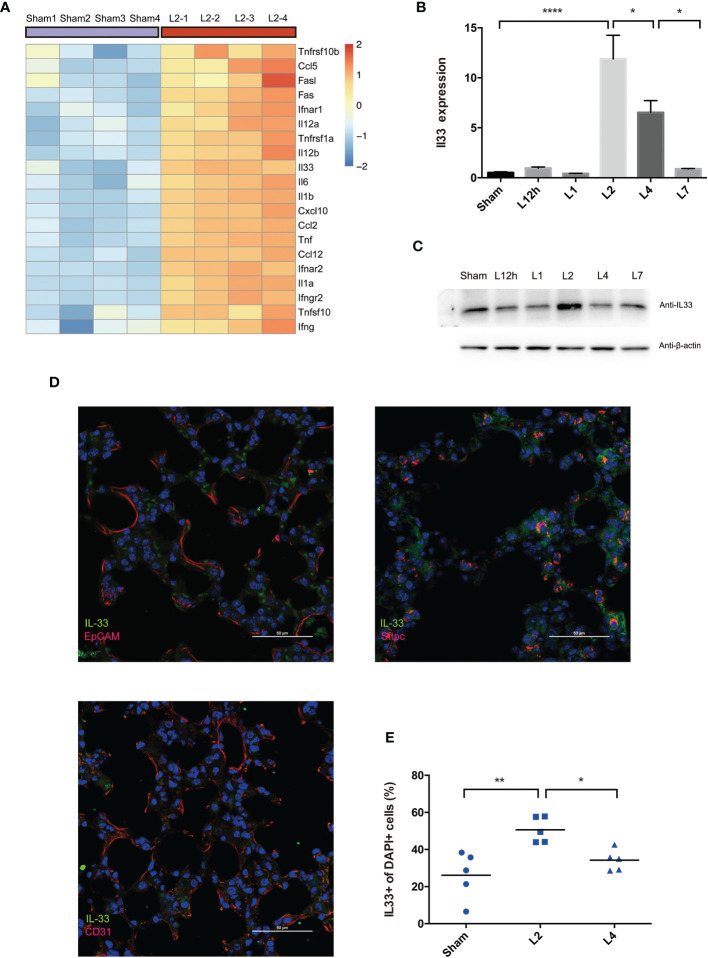
LPS-induced ARDS triggers the release of IL-33 from the inflamed lung. **(A)** Heat map showing the top 20 most upregulated inflammatory cytokines and chemokines in LPS-induced ARDS, identified by mouse RNA-seq analysis. Sham group mice received intratracheal saline instillation. n = 4 mice per group. **(B, C)** IL-33 dynamic variation within days after LPS-induced ARDS. **(B)** Il33 transcripts in the lung were quantified by PCR. **(C)** IL-33 protein was estimated by Western blotting. n = 6 mice per group. **(D)** Colocalization of IL-33+ cells (epithelial cells, AEC2s, and endothelial cells) in the lung was explored by immunofluorescence microscopy. Scale bars represent 50 μm. **(E)** Increase in IL33+ cells upon lung injury. The fraction of IL33+ nuclei (DAPI+ structures) was measured as described in the methods. n = 5 mice per group. For all panels: mean ± S.D. *p ≤ 0.05; **p ≤ 0.01; and ****p ≤ 0.0001 for one-way analysis of variance with the Bonferroni post hoc test for multiple t-tests. A value of p < 0.05 was considered statistically significant.

**Figure 4A** clarified the top 20 upregulated inflammation-associated cytokines. Among these genes, interleukin-33 (IL-33), an alarmin binds to the heterodimeric receptor ST2 (IL-33R, IL1RL1), regulates the release of various proinflammatory cytokines and chemokines, including IL-4, IL-5, IL-9, IL-13, TNF-α, IFN-γ, and IL-1β, and is part of the first line of defense in infection (21). Therefore, in this study, we investigated the possible involvement of IL-33 in LPS-induced ARDS.

qPCR and Western blot analysis co-confirmed that IL-33 transcription peaked on day 2 after LPS administration compared with the sham group and then decreased on day 4 (**Figures 4B, C**).

Immunofluorescence analyses demonstrated the overlap between IL-33 synthesis and pulmonary epithelial expression, revealed the profile of this cytokine’s cellular and intracellular information in the lung. IL-33 localized to the nerve structure (22), epithelium, or vascular endothelium (23, 24). However, our immunofluorescence scanning revealed that IL-33-expressing cells were rare in the epithelium (EpCAM+cells) but readily perceived and most frequent within AEC2s (Sftpc+cells) rather than the vascular endothelium (CD31+cells) (**Figure 4D**). Within 4 days of LPS-induced lung inflammation, IL33+DAPI+cells further proved the upregulation of IL-33 release on day 2, followed by downregulation on day 4 (**Figure 4E**). Although IL33+cells were frequent on day 2, IL33+Sftpc+coexpressing cells were more abundant in the lung on day 4 after LPS administration (**Figure S1**).

We have come to the tentative conclusion that IL-33 is remarkably upregulated in the inflamed lung in LPS-induced ARDS due to increased production and release from the epithelium, especially in AEC2s. Moreover, pulmonary epithelial regeneration was not a short-term effect and was more remarkable on day 4 after lung injury.

### The IL-33:ST2 Axis Impacts Pulmonary Treg Cell Accumulation and Pulmonary Repair

**Figure 5:**
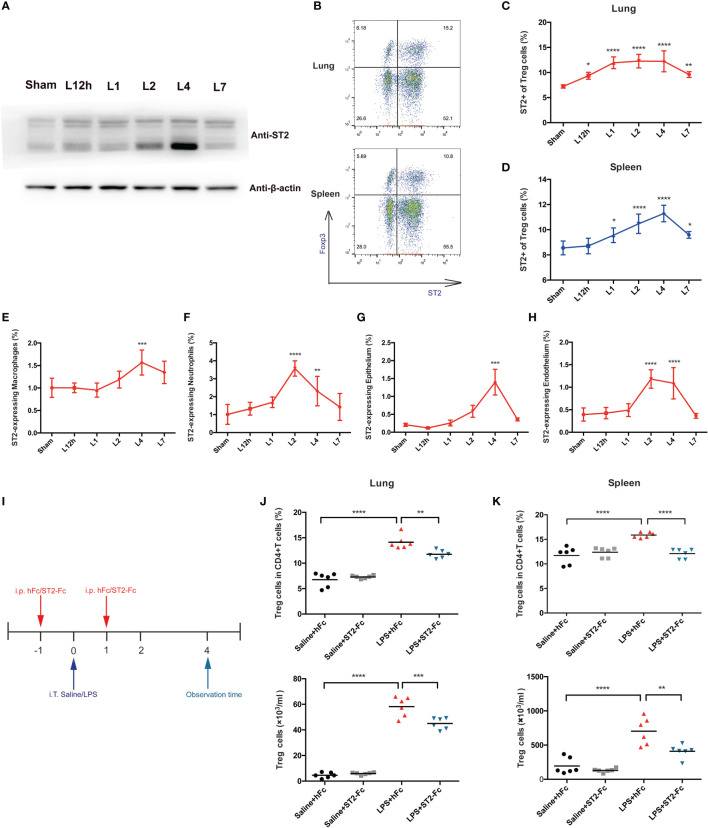
Involvement of the IL-33:ST2 axis in pulmonary Treg cell accumulation. **(A)** A late spike in ST2 expression. BALB/c mice were intratracheally injected with LPS, and at various times, the ST2 protein was detected by Western blotting. The sham group was intratracheally administered saline. **(B–D)** Flow cytometric analysis. Lungs and spleens from mice were prepared at different time points after LPS administration. **(B)** Dot plots of ST2+ Treg in flow cytometry. **(C)** Summary data for the fraction of ST2+ Treg cells in the lung and **(D)** the fraction of ST2+ Treg cells in the spleen within days after LPS administration. **(E, F)** Summary data for the ST2 fraction within different leukocyte populations at various times after LPS administration. **(E)** Fraction of ST2+ macrophages. **(F)** Fraction of ST2+ neutrophils. **(G, H)** Summary data for the ST2 fraction within the epithelium or endothelium at various times after LPS administration. **(G)** Fraction of ST2+ epithelial cells and **(H)** fraction of ST2+ endothelial cells. **(I)** Schematic diagram of the protocol used to establish ST2-blocked mice. BALB/c mice were injected with hFc/ST2-Fc, and 1 day later, intratracheal administration of saline/LPS and hFc/ST2-Fc was performed, with repeated i.p. injection after 1 day. The mice were sacrificed and observed at 4 days after LPS/saline. **(J, K)** Fraction and numbers of Treg cells by flow cytometric analysis, as shown in I, and the observation time was at day 4. **(J)** Treg cells in the lungs. **(K)** Treg cells in the spleen. For all panels: mean ± S.D. *p ≤ 0.05; **p ≤ 0.01; ***p ≤ 0.001; and ****p ≤ 0.0001 for one-way analysis of variance with the Bonferroni post hoc test for multiple t-tests. A value of p < 0.05 was considered statistically significant. n = 6–8 mice per group.

ST2, a receptor of IL-33, was also probed. ST2 protein was also upregulated in the lungs 4 days after the mice received LPS treatment (**Figure 5A**). When deeply exploring the cells that most highly expressed the ST2 protein, we analyzed Treg cells, macrophages, neutrophils, the epithelium, and the endothelium. An elevated fraction of ST2+Treg cells in the lung was first observed 12 h after LPS administration, remained high on day 2 and day 4, and was then reduced on day 7. The fraction of ST2+Treg cells in the spleen peaked on day 4 and showed paralleled variation (**Figures 5B–D**). However, such high expression of ST2 was not a general characteristic of other infiltrated inflammatory cells in inflamed lungs after LPS administration. Although ST2+macrophages, ST2+neutrophils, ST2+epithelial cells, or ST2+endothelial cells mostly revealed significant differences on day 4, ST2 expression was barely observed in macrophages, neutrophils, the epithelium, or the endothelium, the fraction was too low to deserve thoroughly research (**Figures 5E–H**).

To analyze the possible involvement of the IL-33:ST2 axis in Treg cell accumulation and function, we employed ST2-Fc to block ST2. **Figure 5I** shows the schematic diagram. Blocking ST2 impaired Treg cells accumulation in the lung and spleen (**Figures 5J, K**). Then, we explored whether Blocking ST2 retarded Treg cell proliferation, Blocking ST2 did not affect Treg cell proliferation in both the lungs and spleen (data not shown).

**Figure 6:**
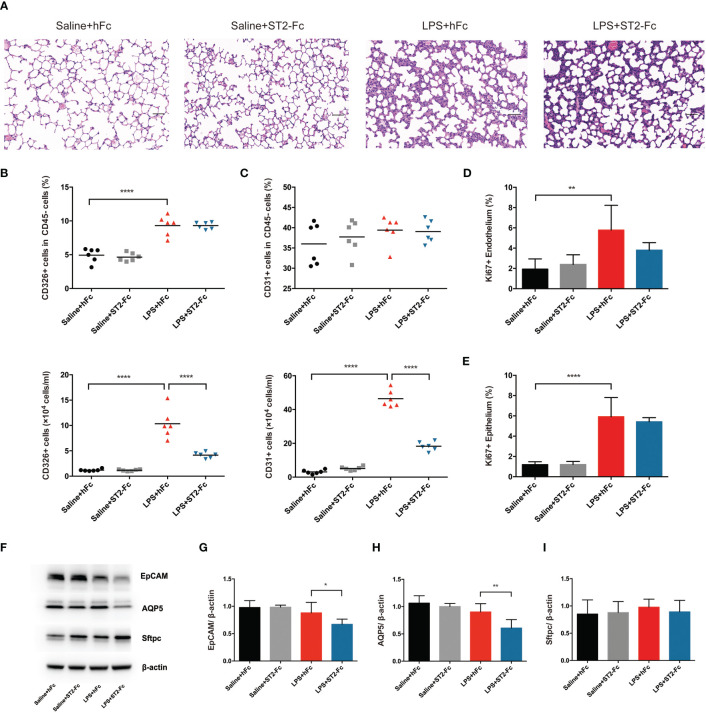
Involvement of the IL-33:ST2 axis in pulmonary regeneration activities. **(A)** Histopathological staining. Representative HE staining of lung sections is shown 4 days after LPS-induced ARDS. Original magnification = 200×, scale bar represents 50 μm. **(B)** Fraction and numbers of epithelial cells (CD45−CD326+CD31− cells) in the lung suspension by flow cytometric analysis. **(C)** Fraction and numbers of endothelial cells (CD45−CD326−CD31+ cells) in the lung suspension by flow cytometric analysis. **(D)** Summary data for proliferation of endothelium (CD45−CD326−CD31+Ki67+ cells) by flow cytometric analysis. **(E)** Summary data for proliferation of the epithelium (CD45−CD326+CD31−Ki67+ cells) by flow cytometric analysis. **(F–I)** Lung epithelium-associated protein. EpCAM, AQP5, and Sftpc protein expression was quantified by Western blotting. The observation time was on day 4. For all panels: mean ± S.D. *p ≤ 0.05; **p ≤ 0.01; and ****p ≤ 0.0001 for one-way analysis of variance with the Bonferroni post hoc test for multiple t-tests. A value of p < 0.05 was considered statistically significant. n = 6 mice per group.

Next, we detected whether blocking ST2 influenced the pulmonary epithelial regeneration. Histopathological staining did not show a significant difference between the LPS+hFC and LPS+ST2-Fc groups (**Figure 6A**). Blocking ST2 indeed damaged the pulmonary epithelium and vascular endothelium, as indicated by the decreased numbers of CD45−CD326+CD31− epithelial cells and CD45−CD326−CD31+ endothelial cells (**Figures 6B, C**), without hampering the proliferation of the pulmonary epithelium or endothelium (**Figures 6D, E**) when the mice were subjected to intratracheal LPS administration.

Epithelial cell adhesion molecule (EpCAM) is a cell surface marker of epithelial cells, especially in stem and progenitor cells, that is expressed at high levels in proliferative cells and at low levels in differentiated cells (25, 26). Subsequently, we elaboratively discovered the alveolar epithelial subset type. Alveolar epithelial cells are comprised of two morphologically and functionally distinct types of cells: alveolar epithelial cells type I (AEC1s) and alveolar epithelial cells type II (AEC2s). Aquaporin 5 (AQP5) is the predominant form expressed in AEC1s, which is related to cellular permeability and regulated cell water movement (17). Surfactant protein C (Sftpc), a specific marker of AEC2s, is a unique transmembrane protein that forms a lipid monolayer at the interface of liquid and air, reduces surface tension along the alveolar epithelium, and mediates effective gas exchange.

ST2 blockade depressed EpCAM and AQP5 protein expression after intratracheal instillation of LPS, although Sftpc protein levels was not showed a distinct decrease (**Figures 6F–I**). This demonstrated that ST2 blockade disrupted pulmonary repair by mitigating endothelial or epithelial regeneration and postponing epithelial recovery.

### Exogenous Supplementation With IL-33 Expands the Treg Cell Population, Enhances Treg Cell Function, and Promotes Lung Regeneration

**Figure 7:**
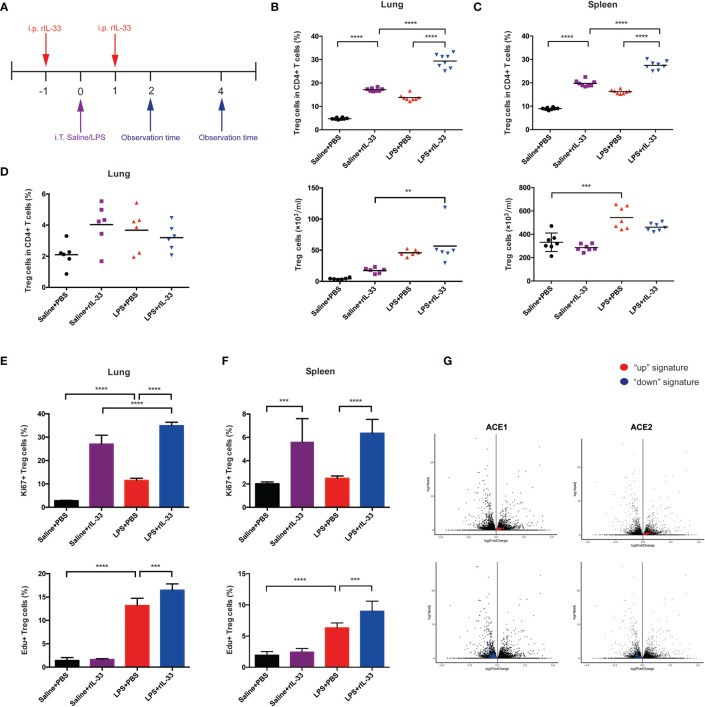
IL-33 activates the Treg cell population in LPS-induced acute respiratory distress syndrome and promotes pulmonary repair. **(A)** Schematic diagram of the protocol for IL-33 supplementation. BALB/c mice were injected with rIL-33, and 1 day later, intratracheal administration of saline/LPS was performed. rIL-33 was repeatedly i.p. injected after 1 day, and the mice were sacrificed and observed at 2 days or 4 days after LPS/saline treatment. **(B, C)** Expansion of the Treg cell population in IL-33-treated mice. BALB/c mice were intratracheally injected with saline or LPS supplemented with rIL-33 in PBS or PBS the day before and after lung injury and were detected 4 days later. **(B)** Expansion fraction and numbers of Treg cells in lungs. **(C)** Expansion fraction and numbers of Treg cell populations in the spleen. **(D)** Expansion of pulmonary Treg cells does not depend on the circulating pool in IL-33-treated mice. Mice were treated with FTY720 or PBS a day prior to LPS-induced lung injury and daily thereafter, and the Treg cells in the lung suspension were measured by flow cytometry 2 days after LPS administration. **(E, F)** Augmentation of Treg cell proliferation in IL-33-treated mice. **(E)** Proliferation of Treg cells in the lung and **(F)** spleen was analyzed via Ki67+ or EdU+ Treg cells by flow cytometry 2 days after saline/LPS administration in addition to IL-33. **(G)** RNA-seq analysis of lung AEC1 or AEC2 regeneration in IL-33-treated mice in the LPS+rIL-33 group vs LPS+PBS group at 4 days. The left upper panel displays the AEC1 “up” signature, and the left lower panel shows the AEC1 “down” signature. The right upper panel displays the AEC2 “up” signature, and the right lower panel shows the AEC2 “down” signature. n = 4 mice per group. For all panels: mean ± S.D. **p ≤ 0.01; ***p ≤0.001; and ****p ≤ 0.0001 for one-way analysis of variance with the Bonferroni post hoc test for multiple t-tests. A value of p < 0.05 was considered statistically significant.

To confirm whether IL-33 could amplify the Treg cell population in inflamed lungs, we administered recombinant IL-33 to mice on the day before and after injury, then analyzed cells by flow cytometry. The experimental schematic is shown in **Figure 7A**. IL-33 indeed augmented the fraction of Treg cells in the lung and spleen (**Figures 7B, C**). However, the Tconv cell population did not change in either the lung or spleen (**Figures S2A–D**).

Then, we disclosed the activity of Treg cells in detail. First, we explored whether IL-33 affects the retention of Treg cells. BALB/c mice were treated with the S1P1 receptor agonist FTY720 at the same time as LPS administration, and pulmonary Treg cells were analyzed by flow cytometry over a 2-day time course. FTY720 was reported to trap T and B cells within lymphoid tissue (27). FTY720 treatment imposed no salient effect on the accumulation of Treg cells (**Figure 7D**). Therefore, the accumulation of pulmonary Treg cells in response to injury seemed to depend more on proliferation from tissue-resident Treg cells. Next, we uncovered whether IL-33 boosts Treg cell proliferation. BALB/c mice were injected with EdU at the time of LPS administration, and 2 days later, EdU+Treg cells from the lung and spleen were quantified; furthermore, we also measured Ki67+Treg cells. According to combined EdU incorporation and Ki67 staining, IL-33 facilitated Treg cell proliferation in lungs and spleen respectively (**Figures 7E, F**).

Whole-lung RNA-sequencing analyses were applied to confirm the effect of IL-33 treatment on pulmonary epithelial regeneration in mice afterwards. Compared with the PBS-treat mice, rIL-33 treated mice showed a strong enrichment of the AEC1 down signature instead of a reduction of the AEC1 up signature after LPS (**Figure 7G**). In addition, rIL-33- and PBS-treated LPS-induced mice showed less apparent skewing of the AEC2 up/down signature (**Figure 7G**), according to previously determined AEC1 and AEC2 up and down signatures (14–16). The balance of AEC2 up/down signatures with IL-33 administration after LPS emphasized AEC2 regeneration. While we did not observe conspicuous AEC1 regeneration on day 4 when the mice were supplemented with IL-33 during ARDS, we could not exclude the possibility that the kinetics of their presence in the lungs at the observed time points were prolonged. Based on the RNA-sequencing evidence, we speculate that IL-33 accelerates pulmonary epithelial repair, especially AEC2 regeneration by expanding Treg cells.

### Transfer of Recombinant Mouse IL-33 Into Treg Depletion Mice Restored Treg Cells and Promoted Lung Epithelial Regeneration

**Figure 8:**
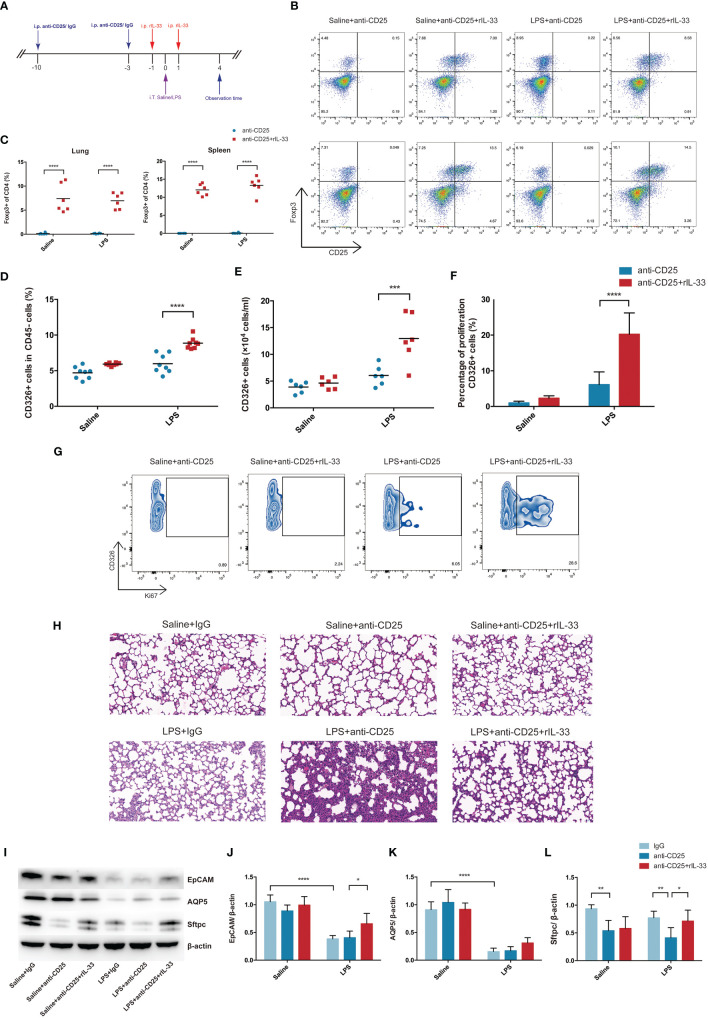
Transfer of recombinant mouse IL-33 into Treg depletion mice restored Treg cells and promoted lung epithelial regeneration. **(A)** Schematic diagrams of IL-33 supplementation in Treg depleted mice during LPS-induced ARDS. Mice were intraperitoneally injection with 100 µg of anti-CD25 antibody or IgG 10 days before LPS exposure and repeatedly treated every 7 days for continuous Treg depletion. Then 1 day before and after LPS administration, rIL-33 (2 mg/mouse) were intraperitoneally injected into mice. Mice were sacrificed at 4 day after LPS administration. **(B)** Cytofluorometric dot plots of Treg cells in Saline+anti-CD25, Saline+anti-CD25+rIL-33, LPS+anti-CD25, LPS+anti-CD25+rIL-33 group. Numbers depict the fraction of CD4+ T cells within the designated gate. The upper panels display the Treg cells in lung, and the lower panels display the Treg cells in spleen. **(C)** Summary data for the percentage of Treg cells in the lung or spleen depicted in **(B)**. **(D)** Summary data for the percentage and **(E)** number of epithelial cells in the lungs. **(F)** Percentage of proliferating epithelial cells in the lung. **(G)** Zebra plots of proliferating epithelial cells percentage in different groups in the lung. **(H)** Histopathological staining. Representative HE staining of lung sections were shown in Saline+IgG, Saline+anti-CD25, Saline+anti-CD25+rIL-33, LPS+IgG, LPS+anti-CD25, LPS+anti-CD25+rIL-33 group. Original magnification = 200×, scale bar represents 50 μm. **(I–L)** EpCAM, AQP5, and Sftpc protein was estimated by Western blotting. For all panels: mean ± S.D. *p ≤ 0.05; **p ≤ 0.01; ***p ≤ 0.001; and ****p ≤ 0.0001 for one-way analysis of variance with the Bonferroni post hoc test for multiple t-tests. A value of p < 0.05 was considered statistically significant. n = 6–8 mice per group.

In order to validate whether transfer of exogenous IL-33 into the Treg-depleted mice will restore the Treg cells population and recover the activity of pulmonary epithelial regeneration, Treg-depleted mice were supplemented with recombinant IL-33 during ARDS, and the schematic diagrams were depicted in **Figure 8A**. There was a prominent restoration of Treg cells both in lung and spleen after recombinant IL-33 supplement into Treg-depleted mice (**Figures 8B, C**). When evaluating the impact of IL-33 ulteriorly, results from flow cytometry indicated IL-33-dependent accumulation of Treg specifically accelerated lung epithelial regeneration. Injection of IL-33 into Treg-depleted mice during ARDS promoted the fraction and number of CD326+ epithelial cells in lung (**Figures 8D, E**), enhanced the proliferation of pulmonary epithelial cells (**Figures 8F, G**). Furthermore, the lung section of HE staining revealed that Treg depletion exacerbated alveolar space collapsion accompanied with excessive alveolar septum or interstitial thickening after LPS. IL-33 supplementation into the Treg-depleted mice mitigated the LPS-induced lung injury and basically regained the normal pulmonary architecture (**Figure 8H**).

EpCAM and AQP5 protein levels were not detected obviously reduced after Treg depletion, whereas Sftpc protein expression were downregulated in Treg depleted mice suffering from LPS-induced ARDS. Critically, after transfer of recombinant IL-33 into Treg depleted mice, EpCAM and Sftpc protein levels were evidently upregulated (**Figures 8I–L**). Therefore, we concluded that exogenous IL-33 supplementation into Treg depleted mice promoted pulmonary epithelial regeneration, and restored the pulmonary alveolar epithelium, especially AEC2s during ARDS.

### IL-33-Dependent Accumulation of Treg Cells Might Mediate Pulmonary Regeneration During LPS-Induced ARDS *via* TGF-β1 Signaling

**Figure 9:**
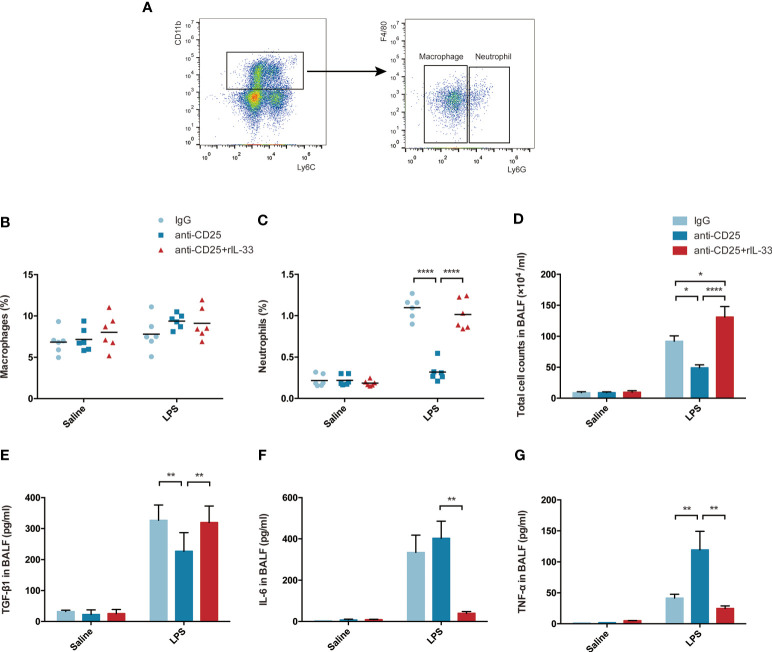
IL-33-dependent accumulation of Treg cells might mediates pulmonary regeneration during LPS-induced ARDS *via* TGF-β1 signaling. **(A–C)** Exogenous IL-33 supplementation did not affect the macrophages, but interfere with neutrophils population in Treg depleted mice after LPS. **(A)** Schematic sorting of macrophages (CD11b+Ly6c+F4/80+Ly6G−) or neutrophils (CD11b+Ly6c+F4/80−Ly6G+) in the lung suspension. The percentage of **(B)** macrophages or **(C)** neutrophils in the lung was detected by flow cytometry. **(D)** Total cell counts in BALF were evaluated by a hemocytometer. **(E)** Secretion of cytokine TGF-β1 in BALF. **(F)** Secretion of cytokine IL-6 in BALF. **(G)** Secretion of cytokine TNF-α in BALF. For all panels: mean ± S.D. *p ≤ 0.05; **p ≤ 0.01; and ****p ≤ 0.0001 for one-way analysis of variance with the Bonferroni post hoc test for multiple t-tests. A value of p < 0.05 was considered statistically significant. n = 6 mice per group.

When delving into the mechanism involved, the fraction of macrophages or neutrophils were assessed by flow cytometry, and related representative cytokine TGF-β1, IL-6, TNF-α, IL-10, IL-21, IL-22, IL-23, IL-9, IFN-γ, IL-1α were examined by bead-based immunoassays. Schematic sorting of macrophages (CD11b+Ly6c+F4/80+Ly6G−) or neutrophils (CD11b+Ly6c+F4/80−Ly6G+) in lung suspensions followed a protocol described in a previous study (28) and were displayed in **Figure 9A**. Although the percentage of macrophages in lung were not showed statistically significant difference, the fraction of neutrophils were declined in Treg-depleted mice after LPS, and increased after exogenous IL-33 supplementation (**Figures 9B, C**). Similarly, the total cell counts in BALF paralleled with the neutrophils variation (**Figure 9D**). Enhanced TGF-β1, IL-6, and TNF-α secretion into the alveolar space were identified after LPS. When LPS-injured mice suffered from Treg depletion, release of TGF-β1 were attenuated, and secretion of IL-6 or TNF-α were obviously intensified. In contrast, after exogenous transfer of IL-33, the level of TGF-β1 were upregulated, and IL-6 or TNF-α were downregulated during ARDS (**Figures 9E–G**). Other cytokines were not of statistical significance (data not shown). Thus, IL-33-dependent accumulation of Treg cells might mediate pulmonary epithelial regeneration in murine model of ARDS *via* TGF-β1 signaling.

## Discussion

Treg cells were recently emphasized in various inflamed tissues, serve as a protective factor in injury, and play an important role in the repair of diverse tissues (8, 29–31). In our study, we verified that inflamed lung injury triggers the aggregation of Treg cells within days. The IL-33:ST2 axis positively modulate the expansion and function of Treg cells, moreover, IL-33-dependent accumulation of Treg cells accelerate pulmonary epithelial regeneration during LPS-induced ARDS. Here, several valuable points merit considerations.

First, in our preliminary study, we have optimized an applicable murine model of ARDS which showed dynamic biological or histopathology changes and virtually completed pulmonary recovery by 4–7 days after lung injury. Importantly, we also identified a successful and effective approach to establish Treg depletion in mice *via* injection of anti-CD25 antibody (19). Therefore, it is convincing to assemble adequate evidence for extending our comprehending of pulmonary regeneration in Treg depletion after ARDS.

Second, when injury occurred, the exogenous danger signal was transduced into an endogenous biochemical reaction, leading to the release of alarmins. IL-33 has been shown to be constitutively expressed in endothelial and epithelial cells *in vivo* (32). However, our data demonstrated that pulmonary IL-33 localized to epithelial cells, especially AEC2s, rather than vascular endothelial cells. Momentously, the identity of the ST2-expressing cell type was somewhat unexpected, ST2 was expressed slightly on epithelial or endothelial cells, even limited on macrophages or neutrophils, but mainly on Treg cells. Although IL-33 expression remained highest on day 2 after lung injury, colocalization was more significant on day 4, paralleled by obvious ST2 expression. Synchronized with colocalization analysis, we speculated it is likely that AEC2s produced and released IL-33, which then combined with ST2 on Treg cells to participate in immunological homeostasis in inflamed lungs.

Third, more recent studies demonstrated IL-33 played an essential role in Treg cell homeostasis (32, 33). In our exploration, administration of IL-33 expanded the Treg cell population by promoting proliferation without intervening retention; when blocking ST2, the number of Treg cells significantly decreased. In addition, exogenous IL-33 supplementation restored Treg cells when mice suffering from Treg depletion. Therefore, through our loss- and gain-of-function study, we validate the IL-33:ST2 axis is the first and most influenced factor affecting the Treg cell population or activity.

Fourth, in addition to vascular endothelium repair, the recovery of alveolar epithelial cells and maintaining the pulmonary epithelial integrity is a fundamental and intrinsic issue in pulmonary repair. AEC2s are reported to be alveolar progenitor cells, are vital in maintaining lung homeostasis and sustaining alveolar integrity *via* release of surfactants, regulation of fluid and ion transport, even restoration of the epithelium by trans-differentiation to AEC1s to initiate pulmonary repair (15). In non-persistent epithelial injury, appropriate AEC2 proliferation and trans-differentiation are elementary to pulmonary repair (34). Knockout of AEC2 in mice obviously restrained injury recovery in acute lung injury (ALI) (35). Consistent with these studies, based on our flow cytometry and western blotting results, we verified that pulmonary epithelial cells, especially AEC2s, were wrecked and their proliferation were undermined in Treg depleted mice, and this is emphasized the significance of Treg cell in maintenance of lung epithelial repair. Then, we exploited IL-33:ST2 axis to facilitate pulmonary repair in mice, after ST2 blockade the number of pulmonary epithelium were diminished; furthermore, pulmonary epithelial cells, especially AEC2s were restored after exogenous IL-33 supplementation into Treg depletion mice. In alliance with IL-33-AEC2 colocalization, it was revealed a close interaction between IL-33-producing AEC2s, Treg cells, and lung epithelial regeneration; IL-33-dependent Treg cells mediated pulmonary epithelial regeneration during ARDS.

Fifth, IL-6 and TNF-α, released by activated neutrophils, are two significant proinflammatory factors used to evaluate inflammation. In our study, associated with highlighted neutrophils, the upregulated levels of IL-6 and TNF-α might collectively indicated that lung regeneration was accompanied by a moderate inflammatory response after IL-33 supplementation. The impact of IL-33 in pulmonary repair will be affected by multiple factors, such as level of inflammation, the type of disease or targeted cells, and the dose of IL-33. Although IL-33 has adverse functions, like as impairments in established immunologic tolerance in the lung (36), even exacerbates sterile liver inflammation (37). Incremental exploration has manifested that proinflammatory cytokines, IL-6, and IL-22, for instance, are critical for wound healing by stimulating keratinocyte migration and collagen deposition. Furthermore, exogenous IL-33 administration during recovery dramatically accelerates epithelial restitution and repair, with concomitant improvement of colonic inflammation in acute colitis (31). When considering the impact of repair, the quality and efficiency of wound repair usually rely on a finely tuned inflammatory response, modulated by the signals released from the damaged epithelium in part (38). Intervention or depression of inflammation in early phase might prolong the normal lung regeneration process.

TGF-β1 was highly expressed by activated T cells, more importantly implicated in Treg-suppressive activity, and controlling inflammatory diseases (39, 40). TGF-β1 are crucial factor secreted by Tregs and pivotal in provoking pulmonary regeneration (19, 41). Concordant with this notion, the strikingly increasing secretion of TGF-β1 were determined after IL-33 supplementation in Treg depleted mice during ARDS in our study. Therefore, we predict that IL-33-dependent accumulation of Tregs likely expedite pulmonary epithelial regeneration in a TGF-β1-dependent manner. More evidence is required in the following study.

Lastly, IL-33 operates as a mechanic-chemical signal and play an important transmittable role between the epithelium, especially AEC2s, and Treg cells. Beyond that, it might also be worth noting that IL-33 improves re-epithelialization and neovascularization, as well as enhances new ECM deposition in diabetic skin wounds (30), and that the involved mechanism is cross-talk between responsive group 2 innate lymphoid cells (ILC2s) and the cutaneous epithelium (30, 42).

The primary targets of IL-33 are likely ILC2 and subsets of Tregs, which are positioned during tissue injury and whose activation creates a reparative state. ILC2 produces large amounts of IL-5 or IL-13 in response to IL-33, and lack of ILC2 results in enhanced inflammation (43). Recently, mounting evidence testified that ILC2-intrinsic IL-33 activation was required for Treg cell expansion to maintain homeostasis (44), and there was tightly interaction between ILC2 and Treg cell (45). Therefore, IL33-producing ILC2 is feasible to accelerate the expansion of pulmonary Treg cells, even to promote their activity in pulmonary epithelial regeneration in the early stage during ARDS.

Irrespective of Treg cells and group 2 innate lymphoid cells (ILC2s), IL-33 also had an impact on other immune cells, including recruited dendritic cells, T helper 1 (Th1) cells, NK cell, invariant natural killer T (iNKT) cell, Th2 cells, promoted cytotoxic T cells, activated macrophages, and elevated neutrophils (46–50). Supported by the recent study that IL-33 motivates Treg cells to suppress innate γδT cell responses in the lungs (51), IL-33 potentially activates other innate immune cells to interact with Treg cells in modulating the activity of pulmonary epithelial repair.

Notably, there is likely to be a complicated network, including exogenous stimuli, immune microenvironment, and crosstalk between innate and adaptive cells, that connects the epithelial regeneration and immune system. Thus, understanding the sophisticated interaction, clarifying the elusive mechanism involved, and finding the optimal intervention will become an area of emerging exploration.

Despite the recent development of comprehensive supportive treatments, such as extracorporeal membrane oxygenation (ECMO) and lung protective ventilation strategies, ARDS still possesses high mortality and poor prognosis. The intensified understanding of the mechanism and identification for new approaches are crucial for addressing the outcome of ARDS. The IL-33:ST2 axis seems to be a promising avenue to utilize in promoting repair of the inflamed lung.

## Data Availability Statement

The original contributions presented in the study are included in the article/**Supplementary Material**. Further inquiries can be directed to the corresponding author.

## Ethics Statement

This study was conducted in accordance with the National Institutes of Health Guide for the Care and Use of Laboratory Animals and the Animal Management Rules of the Chinese Ministry of Health. All experiments were approved by the Animal Care Committee of Peking Union Medical College.

## Author Contributions

WT conducted most of the experiments, acquiring data and writing the manuscript. BZ and XL conducted part of the experiment. CZ and JL analyzed data and provided part of reagents. QM designed the research studies. All authors contributed to the article and approved the submitted version.

## Funding

This work was supported by a grant from the CAMS Innovation Fund for Medical Sciences (CIFMS No. 2017-I2M-1-003).

## Conflict of Interest

The authors declare that the research was conducted in the absence of any commercial or financial relationships that could be construed as a potential conflict of interest.

## Supplementary Material

Supplementary Figure 1 identification of IL-33 localization in AEC2s. Variation in the colocalization of IL-33-producing AEC2s within days: immunofluorescence microscopy of lung sections from the sham group, L2 group, and L4 group. Scale bars represent 50 µm.

Supplementary Figure 2 the Tconv cell population did not change after IL-33 treatment in either the lung or spleen. **(A)** Fraction of Tconv cell in the lung after IL-33 treatment. **(B)** Number of Tconv cell in the lung after IL-33 treatment. **(C)** Fraction of Tconv cell in the spleen after IL-33 treatment. **(D)** Number of Tconv cell in the spleen after IL-33 treatment. Statistical analysis of this figure’s data is shown in **Figure 1**. n = 6 mice per group.
