# Supplementary figures and images for: Interleukin-33-Dependent Accumulation of Regulatory T Cells Mediates Pulmonary Epithelial Regeneration During Acute Respiratory Distress Syndrome
Source: Front Immunol. 2021 Apr 15;12:653803. doi: 10.3389/fimmu.2021.653803 (PMC8082076; doi:10.3389/fimmu.2021.653803)

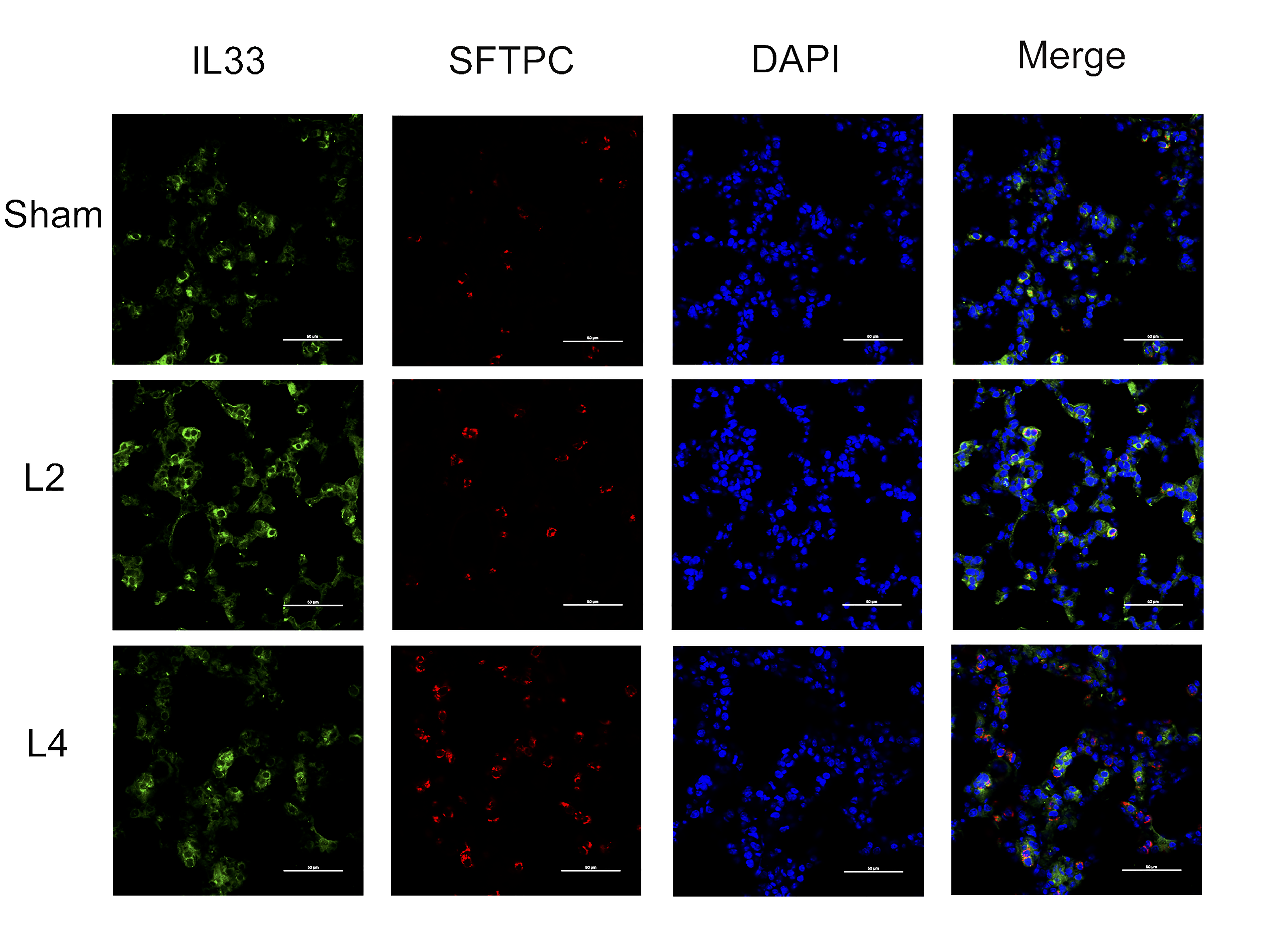

Supplement: Supplementary Figure 1 — identification of IL-33 localization in AEC2s. Variation in the colocalization of IL-33-producing AEC2s within days: immunofluorescence microscopy of lung sections from the sham group, L2 group, and L4 group. Scale bars represent 50 µm. [file Image_1.tif]

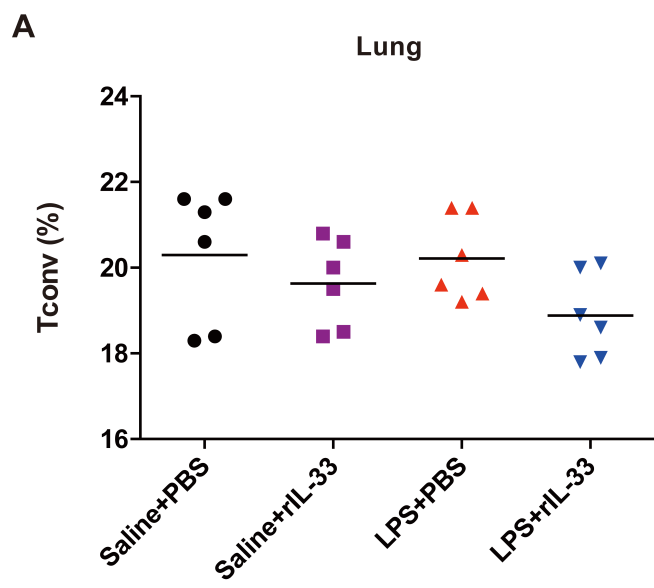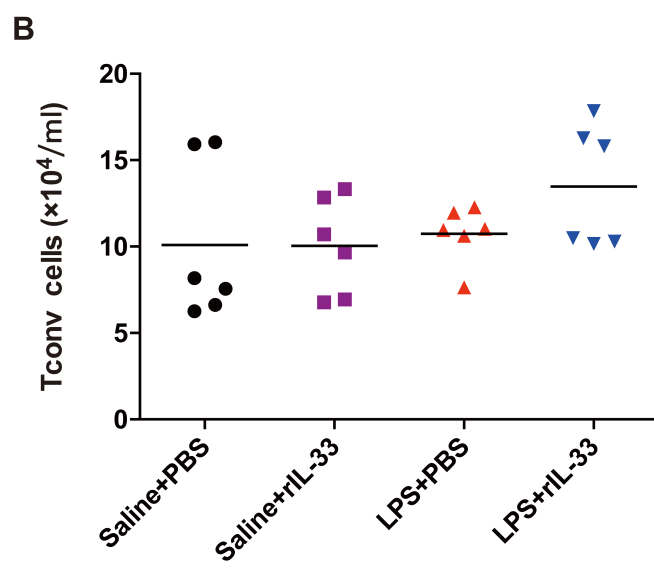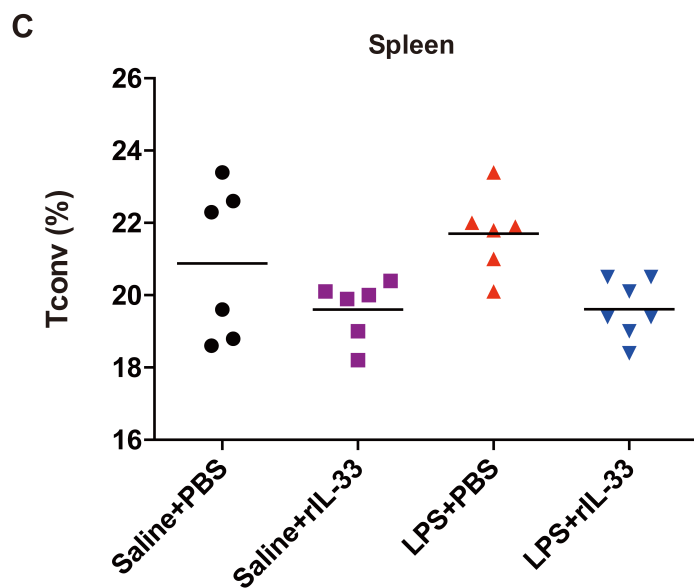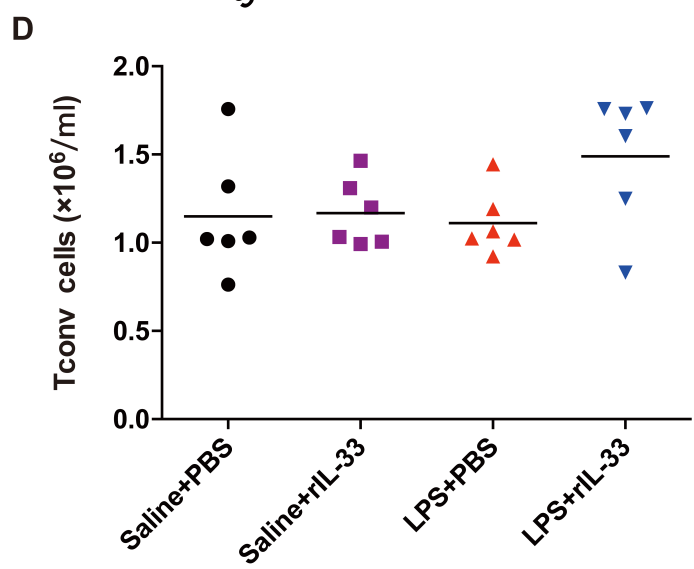

Supplement: Supplementary Figure 2 — the Tconv cell population did not change after IL-33 treatment in either the lung or spleen. (A) Fraction of Tconv cell in the lung after IL-33 treatment. (B) Number of Tconv cell in the lung after IL-33 treatment. (C) Fraction of Tconv cell in the spleen after IL-33 treatment. (D) Number of Tconv cell in the spleen after IL-33 treatment. Statistical analysis of this figure’s data is shown in Figure 1. n = 6 mice per group. [file Image_2.pdf]
